# Transglutaminase 2—a novel inhibitor of adipogenesis

**DOI:** 10.1038/cddis.2015.238

**Published:** 2015-08-27

**Authors:** V D Myneni, G Melino, M T Kaartinen

**Affiliations:** 1 Faculty of Dentistry, McGill University, Montreal, QC, Canada; 2 Department Experimental Medicine and Surgery, University of Rome Tor Vergata, Rome, Italy; 3 Division of Experimental Medicine, Department of Medicine, Faculty of Medicine, McGill University, Montreal, QC, Canada

## Abstract

Differentiation of preadipocytes to lipid storing adipocytes involves extracellular signaling pathways, matrix remodeling and cytoskeletal changes. A number of factors have been implicated in maintaining the preadipocyte state and preventing their differentiation to adipocytes. We have previously reported that a multifunctional and protein crosslinking enzyme, transglutaminase 2 (TG2) is present in white adipose tissue. In this study, we have investigated TG2 function during adipocyte differentiation. We show that TG2 deficient mouse embryonic fibroblasts (*Tgm2*−/− MEFs) display increased and accelerated lipid accumulation due to increased expression of major adipogenic transcription factors, *PPARγ* and *C/EBPα*. Examination of Pref-1/Dlk1, an early negative regulator of adipogenesis, showed that the Pref-1/Dlk1 protein was completely absent in *Tgm2*−/− MEFs during early differentiation. Similarly, *Tgm2*−/− MEFs displayed defective canonical Wnt/β-catenin signaling with reduced β-catenin nuclear translocation. TG2 deficiency also resulted in reduced ROCK kinase activity, actin stress fiber formation and increased Akt phosphorylation in MEFs, but did not alter fibronectin matrix levels or solubility. TG2 protein levels were unaltered during adipogenic differentiation, and was found predominantly in the extracellular compartment of MEFs and mouse WAT. Addition of exogenous TG2 to *Tgm2*+/+ and *Tgm2*−/− MEFs significantly inhibited lipid accumulation, reduced expression of *PPARγ* and *C/EBPα*, promoted the nuclear accumulation of β-catenin, and recovered Pref-1/Dlk1 protein levels. Our study identifies TG2 as a novel negative regulator of adipogenesis.

The prevalence of obesity is steadily increasing globally and is recognized as a major risk factor for diabetes, heart disease and certain cancers.^1, 2, 3^ During excess energy intake adipose tissue expands to store extra lipids. This expansion initially occurs via an increase in the size of existing adipocytes (hypertrophy) which is followed by an increase in adipocyte number via proliferation of preadipocytes (hyperplasia) and their differentiation into mature adipocytes (adipogenesis).^3^ Impaired adipogenesis and adipose tissue function are associated with the development of metabolic complications in obesity, such as the development of type 2 diabetes.^4, 5^

Adipogenesis involves conversion of spindle-shaped preadipocytes to round lipid filled adipocytes, this morphological change requires conversion of filamentous actin to cortical actin^6, 7^ which is associated with remodeling of extracellular matrix (ECM) fibronectin (FN) matrix to laminin-rich matrix. Adipogenesis is regulated by various factors that can either promote or inhibit adipogenesis. Many of these factors regulate ECM components and cytoskeletal tension, some of the factors or proteins which maintain preadipocyte state and act as inhibitors during early phase of adipogenesis include Wnt/β-catenin signaling, Pref-1/Dlk1, RhoA and ROCK kinases. These factors are amongst those which determine whether preadipocytes will be in quiescence, or undergo proliferation and differentiate to adipocytes.^8, 9^

In our previous work we have identified two members of transglutaminase (TG) enzyme family, Factor XIII-A (FXIII-A) and transglutaminase 2 (TG2), in white adipose tissue (WAT).^10^ TGs are enzymes with ability to form isopeptide bonds between glutamine residue of one protein to a lysine residue of another protein by transamidation reaction.^11, 12, 13^ TGs can also have functions that do not involve their transamidase activity.^11^ In our recent work, we have shown that FXIII-A is responsible for the transamidase/crosslinking activity during adipocyte differentiation. In 3T3-L1 adipocyte and mouse embryonic fibroblasts (MEFs) cultures, FXIII-A crosslinking activity increased plasma FN assembly into preadipocyte matrix which promoted preadipocyte proliferation. Inhibition of TG activity of FXIII-A in these cultures resulted in increased adipocyte differentiation.^10^ The role of TG2 in adipogenesis remained unaddressed.

TG2 is the most ubiquitous of the TG family members and expressed in many tissues such as bone, cartilage, kidney, colon, liver, heart, lung, spleen, blood and nervous tissue.^11, 13, 14, 15, 16^ TG2 is expressed by many cell types such as osteoblasts,^17^ chondrocytes,^18, 19^ mesenchymal stem cells (MSCs),^19, 20^ neuronal and glial cells,^11, 12, 21^ phagocytes, monocytes, neutrophils and T-cells^11, 13, 22, 23^ and pancreatic β-cells.^24^ TG2 has been implicated in various biological functions including cell differentiation and maturation, cell morphology and adhesion, ECM stabilization, cell death, inflammation, cell migration and wound healing.^11, 12, 13^ TG2 is present in both extracellular and intracellular compartments of the cell. In the extracellular compartment, TG2 can be found on the cell surface and in the ECM. In the intracellular compartment, TG2 is mostly cytosolic but also found on the plasma membrane, in the nuclear membrane and in mitochondria.^11, 12, 13, 25^ Dysregulation of TG2 function(s) has been implicated in pathogenesis of celiac disease,^11, 13, 26^ diabetes,^24^ neurodegenerative disorders such as Huntington's, Alzheimers's and Parkinson's disease^11, 12, 21^ as well as inflammatory disorders and cancer.^11^

In this study, we have used MEFs from TG2 wild-type (*Tgm2*+/+) and TG2 deficient mice (*Tgm2*−/−) to address the potential role of TG2 during adipocyte differentiation. We report that TG2 deficiency results in accelerated and increased adipogenesis in MEFs due to increased expression of adipogenic transcription factors PPARγ and C/EBPα. We further examined the role of TG2 in several anti-adipogenic pathways and demonstrate that TG2 regulates adipogenesis via multiple factors – these include Pref-1/Dlk1 expression and modulation of Wnt/β-catenin signaling, ROCK kinase activity and Akt signaling.

## Results

### *Tgm2*−/− MEFs show increased and accelerated adipocyte differentiation

**Figure 1:**
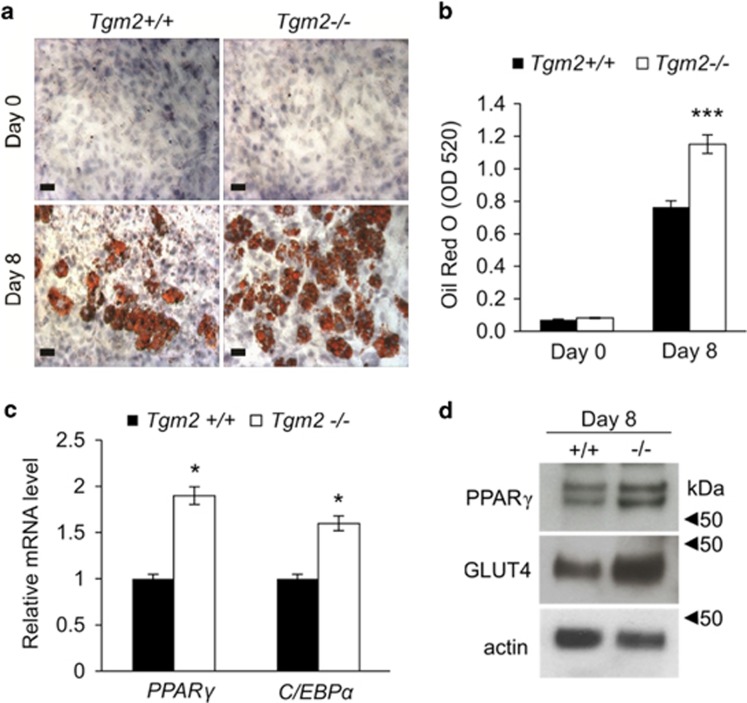
*Tgm2*−/− MEFs show enhanced adipogenesis. (**a**) *Tgm2+/+* and *Tgm2*−/− MEFs were subjected to adipogenic differentiation and their ability to accumulate lipids was assessed on days 0 and 8 by Oil Red O staining. Cells were counter stained with hematoxylin. Increases lipids are visible in *Tgm2*−/− MEFs on day 8. Scale bar equals 70 *μ*m. (**b**) Quantification of Oil Red O cultures on days 0 and 8 show significantly increases lipid accumulation to *Tgm2*−/− MEFs compared with *Tgm2+/+* MEFs. Results are mean values±SEM (n=3). ***P<0.001. (**c**) mRNA expression analysis of *Pparγ* and *Cebpα* from *Tgm2+/+* and *Tgm2*−/− MEFs on day 8 shows a significant increase in *Tgm2*−/− MEFs. The relative quantity of mRNA expression was normalized to 18 S. Error bars±SD (*n*=3), *P<0.05. (**d**) Western blot analysis of total cell lysate of *Tgm2*−/− and *Tgm2*+/+ MEFs on day 8, show increased PPARγ protein and its downstream target GLUT4 in *Tgm2*−/− MEFs; actin used as loading control

Our previous work identified two TG enzymes, FXIII-A and TG2, in mouse WAT and in the 3T3-L1 preadipocyte cell line, and identified FXIII-A as a regulator of preadipocyte proliferation.^10^ In this study, we investigated the role of TG2 in adipogenesis by using TG2 deficient and wild-type MEFs as a model, and examined *Tgm2*+/+ and *Tgm2*−/− MEFs capacity to differentiate into adipocytes under adipogenic conditions. Oil Red O staining for lipid and quantification on day 8 of adipogenesis, shows a 1.5-fold increase in adipose conversion in *Tgm2*−/− MEFs compared with *Tgm2*+/+ cells (Figures 1a and b). Increased adipogenesis was associated with an increase in mRNA expression levels of main adipogenesis transcription factors, *Pparγ* and *Cebpα Tgm2*−/− MEFs showing a 1.8-fold and 1.5-fold increase, respectively, compared with *Tgm2*+/+ MEFs on day 8 (Figure 1c). The increase in the transcription factor mRNA in *Tgm2*−/− MEFs was also associated with an increase in PPAR*γ* protein levels and increased production of its downstream target GLUT4 (Figure 1d).

**Figure 2:**
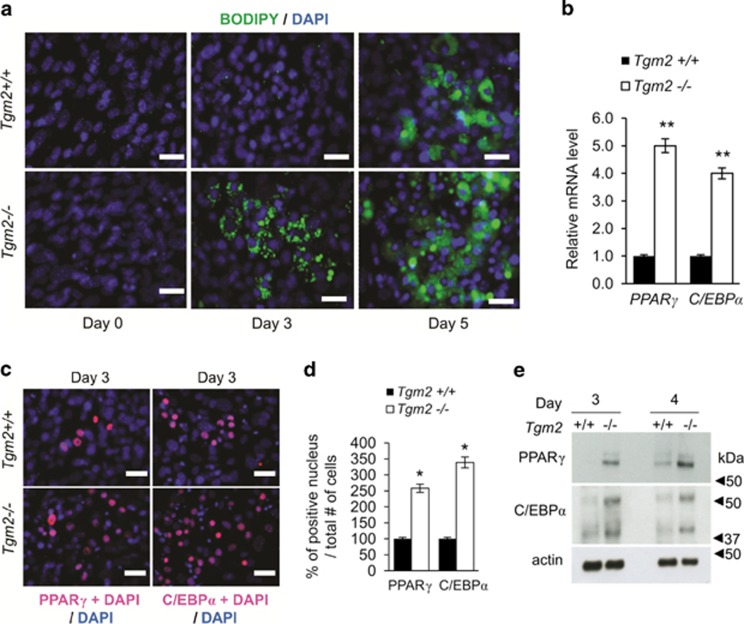
*Tgm2*−/− MEFs show accelerated adipogenesis. (**a**) Immunofluorescence staining for lipid with Bodipy 493/503 (green) during differentiation of *Tgm2+/+* and *Tgm2*−/− MEFs. Lipid accumulation is visible on day 3 in *Tgm2*−/− MEFs compared with *Tgm2+/+* MEFs which show lipids only on day 4–5 onwards. Nuclei were visualized with DAPI (blue). Scale bar equals 50 *μ*m. (**b**) mRNA expression of *Pparγ* and *Cebpα* from *Tgm2+/+* and *Tgm2*−/− MEFs on day 3, show a significant increase in *Tgm2*−/− MEFs. The relative quantity of mRNA expression was normalized to 18 S. Error bars±SD (*n*=3), **P<0.01. (**c**) Nuclear translocation of PPARγ and C/EBPα (colocalization with DAPI in pink) in *Tgm2+/+* and *Tgm2*−/− MEFs on day 3 show increased activation of transcription factors. Nuclei stained with DAPI (blue). Scale bar equals 50 *μ*m. (**d**) Quantification of PPARγ and C/EBPα positive nucleus per total number of cells in *Tgm2+/+* and *Tgm2*−/− MEFs on day 3 shows a dramatic and significant increase in *Tgm2*−/− MEFs. Error bars±SEM (*n*=3), *P<0.05. (**e**) Western blot analysis of PPARγ and C/EBPα in total cell lysates on days 3 and 4 show increased PPARγ and C/EBPα protein levels in *Tgm2*−/− MEFs; actin used as loading control

Time course analysis of lipid droplet accumulation in cells during early differentiation on days 0, 3 and 5, show that *Tgm2*−/− MEFs accumulate lipids earlier on day 3 compared with *Tgm2*+/+ MEFs that show lipids on days 4–5 (Figure 2a). Accelerated adipogenesis was associated with an increase in mRNA expression levels of *Ppar*γ and *Cebp*α*; Tgm2*−/− MEFs showed a 5-fold and 4-fold increase, respectively compared with *Tgm2*+/+ MEFs on day 3 (Figure 2b). Increase in mRNA expression was also accompanied by significantly increased PPARγ and C/EBPα positive nuclei in *Tgm2*−/− MEFs compared with *Tgm2*+/+ MEFs on day 3 indicative of their increased nuclear translocation and thus activation (Figures 2c and d). Western blot analysis of PPARγ and C/EBPα show that both are upregulated in *Tgm2*−/− MEFs compared with *Tgm2*+/+ MEFs. PPARγ was detected in *Tgm2*−/− MEFs but not *Tgm2*+/+ MEFs on day 3, and PPARγ was detected in *Tgm2*+/+ MEFs by day 4 supporting the accelerated adipogenesis seen in *Tgm2*−/− MEFs (Figure 2e). These results indicate that TG2 is a negative regulator of adipogenesis.

### TG2 is critical for Pref-1 protein expression

**Figure 3:**
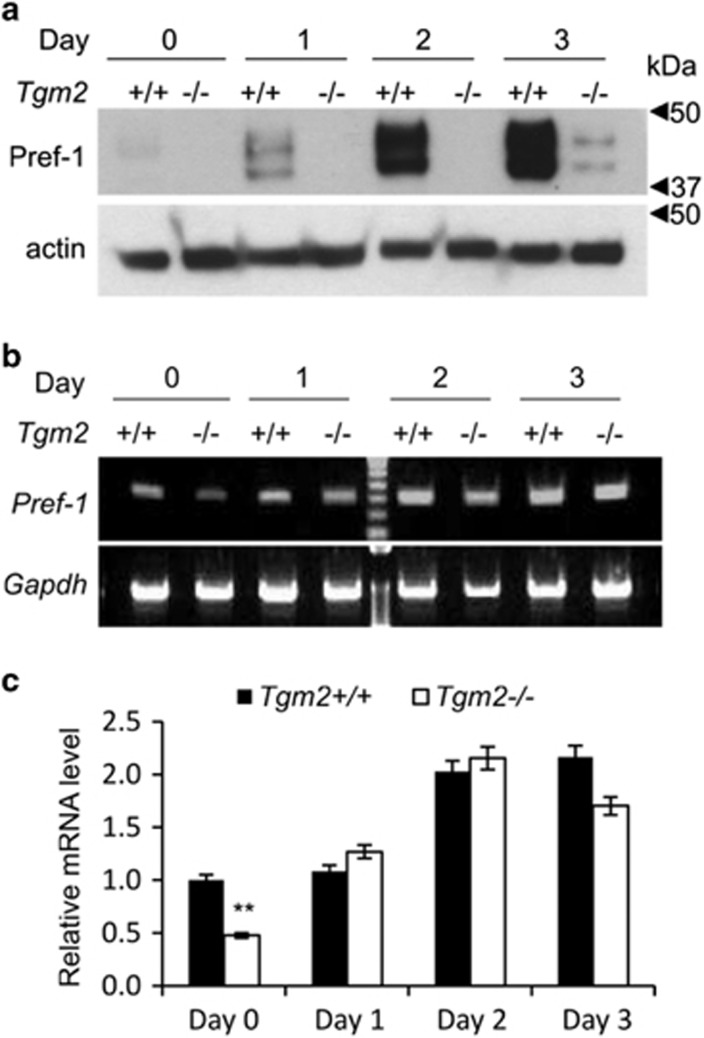
Pref-1 protein and mRNA production is compromised in *Tgm2*−/− MEFs. (**a**) Western blot analysis of Pref-1f in total cell lysates of *Tgm2*−/− and *Tgm2*+/+ MEFs during early differentiation on days 0–3 show almost complete absence of the protein. Actin was used as loading control. (**b**) mRNA expression of *Pref-*1 by RT-PCR in the cells shows that on day 0 Pref-1 mRNA is lower in *Tgm2*−/− compared with *Tgm2*+/+ MEFs; however, on day 3 the difference is no longer observed. This mRNA does not appear to translate into protein as per Western blot analysis. *Gapdh* used as loading control. (**c**) Quantification of mRNA expression of Pref-1 (**b**), shows significantly reduced of Pref-1 on day 0 in *Tgm2*−/− compared with *Tgm2*+/+ MEFs. Pref-1 expression was similar in both *Tgm2*−/− and *Tgm2*+/+ MEFs after initiation of differentiation (day 1). Error bars±SEM (*n*=3), **P<0.01

Because of the accelerated adipogenesis in *Tgm2*−/− MEFs, we examined Pref-1/Dlk-1 expression levels in these cells. Pref-1 inhibits adipogenesis during the early phase of differentiation, and Pref-1 downregulation coincides with upregulation of C/EBPα and PPARγ.^27, 28, 29^ Examination of Pref-1 protein levels in total cell lysate reveals a dramatic loss of Pref-1 protein in *Tgm2*−/− MEFs compared with *Tgm2*+/+ MEFs. Only very low levels are detected at day 3 of adipogenesis (Figure 3a). mRNA expression of *Pref*-1 (Figures 3b and c) on day 0 was significantly lower in *Tgm2*−/− MEFs, but no significant difference was observed between *Tgm2*−/− and *Tgm2*+/+ MEFs after the initiation of adipocyte differentiation. However, this similar mRNA expression did not result in an increase in Pref-1 protein expression suggesting that TG2 regulates mainly Pref-1 protein production.

### TG2 is required for β-catenin nuclear translocation

**Figure 4:**
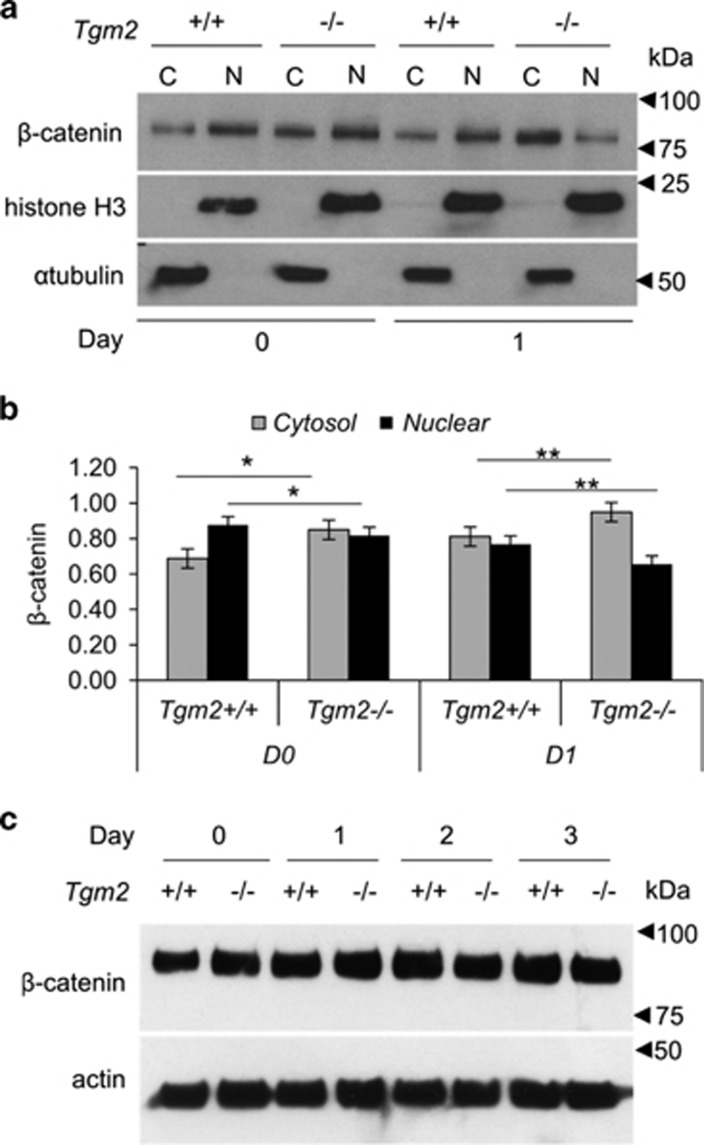
*Tgm2*−/− MEFs show decreased β-catenin nuclear translocation. (**a**) Western blot analysis of β-catenin levels in cytosol (C) and nuclear (N) fractions of *Tgm2+/+* and *Tgm2*−/− MEFs on days 0 and 1. α-Tubulin and histone H3 were used as cytosolic and nuclear loading controls, respectively. (**b**) Quantification of Western blots shows significantly reduced nuclear translocation of β-catenin in *Tgm2*−/− MEFs compared with control cells before (day 0) and after initiation of differentiation (day 1). Error bars±SEM (*n*=3), *P<0.05; **P<0.01. (**c**) Western blot analysis for β-catenin levels in total cell lysates of *Tgm2+/+* and *Tgm2*−/− MEFs from days 0–3 show no changes; actin used as a loading control

Because of the links of TG2 to canonical Wnt/β-catenin signaling^30^ and its inhibitory role in adipogenesis, and PPARγ and C/EBPα expression,^31, 32^ we examined canonical Wnt/β-catenin pathway to see if it is affected in *Tgm2*−/− MEFs. Examination of β-catenin nuclear translocation—a hallmark of Wnt signaling activation in cells—show that *Tgm2*−/− MEFs have significantly decreased β-catenin levels in the nucleus and increased in cytosol compared with *Tgm2*+/+ cells (Figures 4a and b). Total β-catenin levels were not altered (Figure 4c). These results indicate that TG2 inhibits early phase of adipogenesis by regulating Pref-1 production and β-catenin signaling.

### TG2 is predominantly extracellular during early adipogenesis

**Figure 5:**
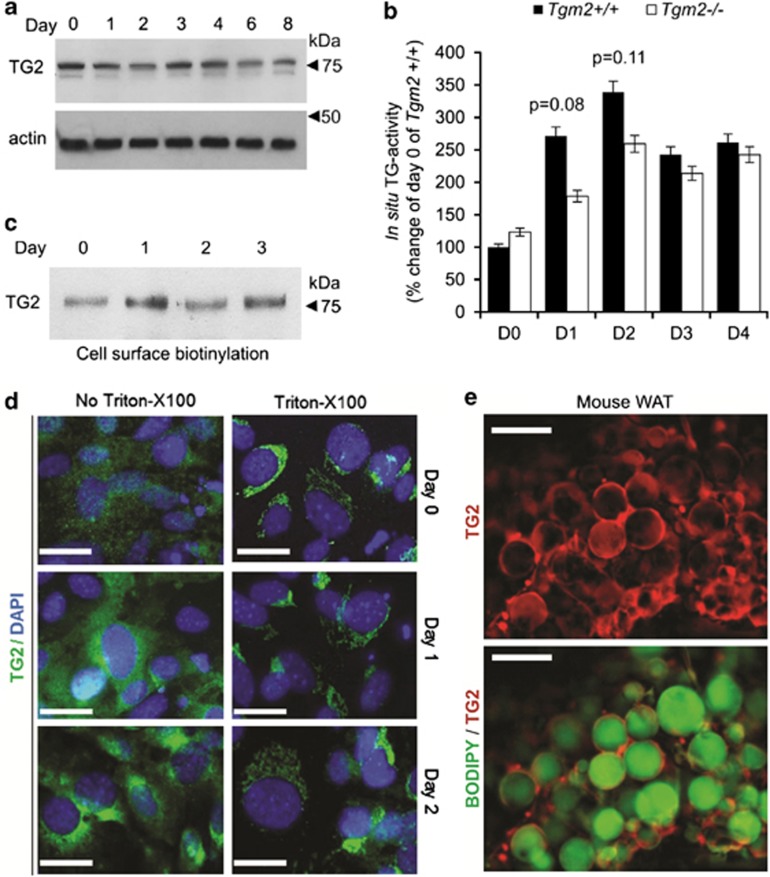
TG2 levels and location during early adipogenesis and in WAT. (**a**) Western blot analysis of total cell lysate from *Tgm2+/+* MEFs during adipocyte differentiation. TG2 levels remain constant with no major fluctuations during differentiation. Actin used as loading control. (**b**) Transamidase activity in *Tgm2*−/− and *Tgm2*+/+ MEFs during adipogenesis was assessed *in situ* using 5-(biotinamido) pentylamine as an activity probe. Graph displayed is biotin detection in cells and the activity is normalized to TG-activity on day 0 of *Tgm2+/+* MEFs (set for 100%). Results are mean values±SEM (*n*=3). (**c**) Western blot analysis of cell surface biotinylated protein extract for TG2 protein levels in *Tgm2+/+* MEFs during adipocyte differentiation. TG2 levels on cell surface increase with initiation of differentiation (day 1). (**d**) Immunofluorescence staining of TG2 (green) during early differentiation of *Tgm2+/+* MEFs. Nuclei were stained with DAPI (blue). Cells not treated with Triton-X100 show the extracellular distribution of TG2; Triton X-100 permeabilized cells show intracellular distribution; Scale bar equals 100 *μ*m. (**e**) Immunofluorescence staining of whole-mount mouse white adipose tissue (WAT) showing distribution of TG2 (red) and lipids (Bodipy 493/503, green) in the tissue; TG2 is mainly extracellular; Scale bar equals 50 *μ*m

To gain mechanistic insight into the inhibitory effect of TG2 during adipogenesis, we examined the TG2 protein levels, *in situ* TG-activity and cellular localization in *Tgm2*+/+ MEFs during the course of differentiation and its location in WAT. Figure 5a, shows that TG2 total protein levels did not markedly change during adipogenesis. TG-activity, measured *in situ* by growing the MEFs with 5-(biotinamido)pentylamine, showed no significant change in *Tgm2*−/− MEFs compared with *Tgm2*+/+ MEFs during early adipocyte differentiation, suggesting that the lack of TG-activity is not the cause of inhibitory effects seen in *Tgm2*−/− MEFs (Figure 5b). It has been shown that in airway epithelial cell lines, TG2 crosslinks PPARγ to a higher molecular weight form between 72 and 250 kDa, which contributes to change in the monomer (55 kDa) levels.^33^ Western blot analysis for PPARγ after reduced and nonreduced SDS-PAGE condition of total cell lysate on day 8 showed no higher molecular weight products of PPARγ (Supplementary Figure S1) demonstrating that crosslinking is not involved in its regulation. Cell surface biotinylation experiments (Figure 5c) and immunofluoresence staining (Figure 5d) of TG2 in *Tgm2*+/+ MEFs that were not permeabilized by Triton-X100 shows that TG2 is found in the extracellular space and increased cell surface expression on day 1 of differentiation. Whole-mount immunofluorescence staining of mouse epidydimal WAT confirms that TG2 is mainly present in the extracellular space of adipose tissue (Figure 5e).

### Exogenous TG2 inhibits adipogenesis and increases β-catenin nuclear translocation, and Pref-1 protein expression

**Figure 6:**
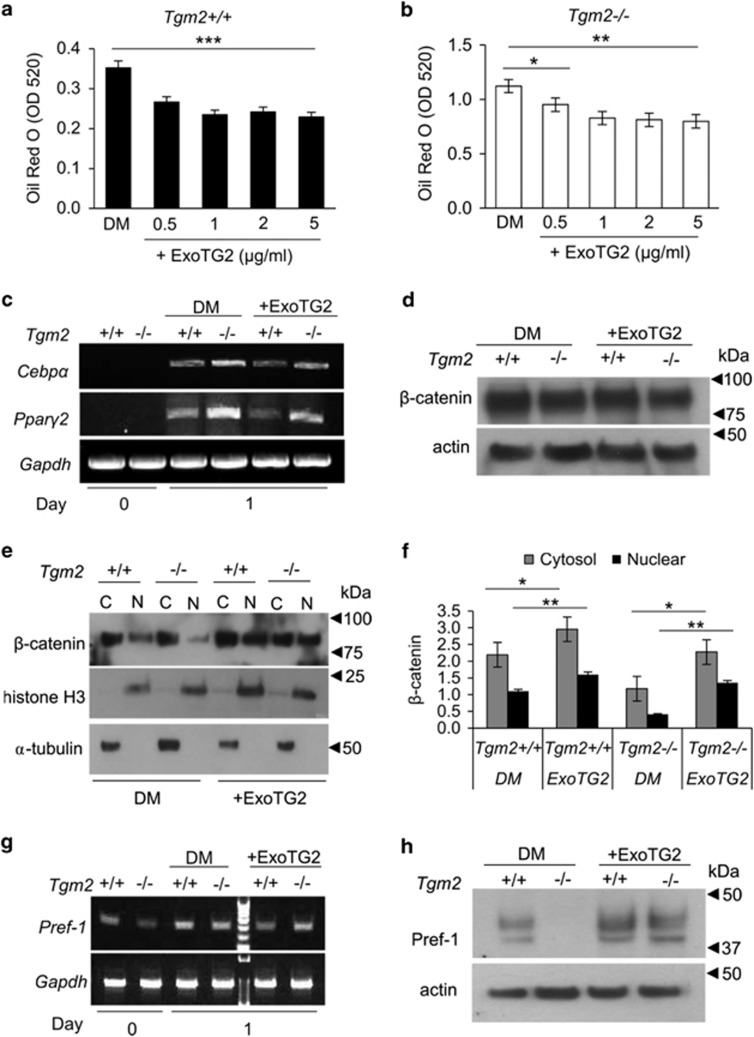
Exogenous, extracellular TG2 inhibits adipogenesis and activates β-catenin signaling and recovers Pref-1 protein levels. (**a**, **b**) *Tgm2+/+* and *Tgm2*−/− MEF cultures were treated with increasing concentrations (0.5–5 *μ*g/ml) of exogenous TG2 (ExoTG2) from days 0 to 8. Graphs show quantification of Oil Red O staining on day 8. Exogenous TG2 was able to reduce lipid accumulation in a significant manner in both *Tgm2+/+* and *Tgm2*−/− MEFs. Results are mean values±SEM (*n*=3). ***P<0.001. *P<0.05; **P<0.01. (**c**) mRNA expression of *Pparγ* and *Cebpα* in *Tgm2+/+* and *Tgm2*−/− MEFs on days 0 and 1 with or without ExoTG2 (5 *μ*g/ml); DM-differentiation medium. A reduced expression was observed with ExoTG2. (**d**) Western blot analysis of total β-catenin levels in total cell lysate of *Tgm2+/+* and *Tgm2*−/− MEFs on day 1 with or without ExoTG2 (5 *μ*g/ml) show no difference; actin used as a loading control. (**e**, **f**) Western blot analysis and quantification of β-catenin levels in cytosolic (**c**) and nuclear (N) fractions of *Tgm2+/+* and *Tgm2*−/− MEFs on day 1 with or without ExoTG2 (5 *μ*g/ml). Normalization was done to loading controls α-tubulin and histone H3. *Tgm2*−/− MEFs show significantly increased levels of β-catenin in the nucleus. Error bars±SEM (*n*=3), *P<0.05; **P<0.01. (**g**) mRNA expression of *Pref*-1 in *Tgm2+/+* and *Tgm2*−/− MEFs on days 0 and 1 with or without ExoTG2 (5 *μ*g/ml). (**h**) Western blot analysis of total cell lysate for Pref-1 in *Tgm2+/+* and *Tgm2*−/− MEFs on day 1 with or without ExoTG2 (5 *μ*g/ml). ExoTG2 treatment recovered Pref-1 protein levels in *Tgm2*−/− MEFs

To investigate if extracellular TG2 regulates adipogenesis, exogenous TG2 enzyme (ExoTG2) was added to *Tgm2*+/+ and *Tgm2*−/− MEF cultures during differentiation. The addition of ExoTG2 caused a significant decrease in lipid accumulation in both *Tgm2*+/+ and *Tgm2*−/− MEFs (Figures 6a and b). Lipid accumulation was reduced by 23–35% in *Tgm2*+/+ MEFs and 16–29% in *Tgm2*−/− MEFs with concentrations ranging from 0.5 to 5 *μ*g/ml. Reduced adipogenesis by ExoTG2 was also associated with reduced *Ppar*γ and *Cebp*α expression in *Tgm2*+/+ and *Tgm2*−/− MEFs (Figure 6c; Supplementary Figure S2A,B). The total β-catenin levels were not altered by ExoTG2 compared with controls (Figure 6d), but significantly increased nuclear β-catenin in *Tgm2*−/− MEFs (Figures 6e and f). *Pref*-1 mRNA expression was not altered by ExoTG2 in *Tgm2*−/− MEFs (Figure 6g; Supplementary Figure S2C). However, ExoTG2 completely recovered the Pref-1 protein levels in *Tgm2*−/− MEFs (Figure 6h). These results suggest that extracellular TG2 inhibits adipogenesis and regulates Pref-1 protein production, but not mRNA expression.

### *Tgm2*−/− MEFs show decreased ROCK kinase activity and increased Akt phosphorylation—no changes in FN matrix levels

**Figure 7:**
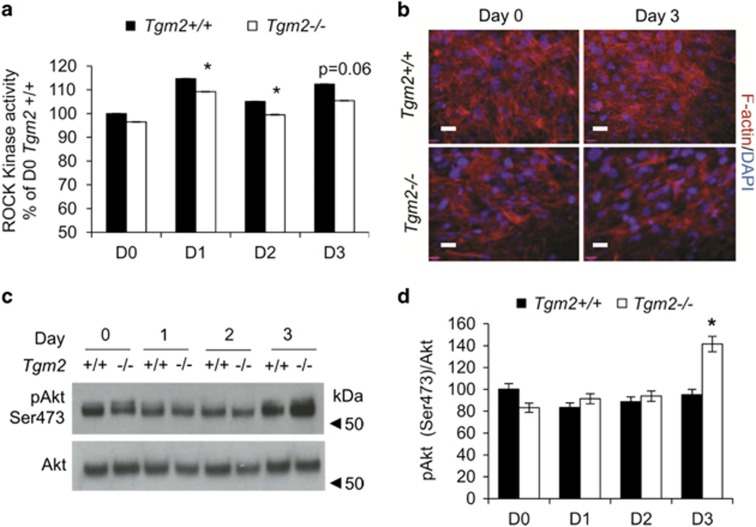
*Tgm2*−/− MEFs display reduced ROCK kinase activity, actin fibers and increased Akt phosphorylation. (**a**) Microplate ROCK kinase activity of *Tgm2+/+* and *Tgm2*−/− MEFs in total cell lysate during differentiation show moderate but significant decrease in *Tgm2*−/− cells on days 1 and 2. Error bars±SEM (*n*=3), *P<0.05. (**b**) Immunofluorescence staining of of *Tgm2+/+* and *Tgm2*−/− MEFs for F-actin on days 0 and 3. A moderate decrease in actin stress fibers is observed. Nuclei are stained with DAPI (blue). Scale bar 200 *μ*m. (**c**, **d**) Western blot analysis and quantification of pAkt (Ser473) and total Akt in MEF cell lysates from day 0–3. An increase in Akt phosphorylation is seen on day 3 in *Tgm2*−/− MEFs compared with *Tgm2+/+* MEFs. Results are mean values±SEM (*n*=3), *P<0.05

In search other potential anti-adipogenic pathways that TG2 may regulate we considered the facts that adipogenesis involves major cytoskeletal changes to accommodate to lipid storage. Given the function of TG2 in regulating actin cytoskeleton via RhoA-ROCK signaling^34^ and the role of ROCK as an inhibitor of adipogenesis^35^ we analyzed ROCK activity during early differentiation. Data shows a moderate but significant downregulation of ROCK activity in *Tgm2*−/− MEFs on days 1 and 2 (Figure 7a). Examination of actin stress fibers, that are regulated by ROCK kinase,^36^ by immunofluorescence show reduced F-actin network in *Tgm2*−/− MEFs compared with *Tgm2+/+* MEFs (Figure 7b). It is known that inhibition of ROCK enhances Akt signaling, which has a crucial promoting role in adipocyte differentiation and PPARγ regulation.^35, 37, 38^ Figures 7c and d shows a significant upregulation of Akt phosphorylation on day 3 in *Tgm2*−/− MEFs compared with *Tgm2+/+* MEFs. The effect of TG2 deficiency on cytoskeleton is not mediated by FN matrix levels as *Tgm2*−/− MEFs assembled normal FN matrix and showed no changes in the amounts of FN in DOC-soluble or DOC-insoluble fractions (Supplementary Figure S3). mRNA expression of total and cellular FN (EDA-FN or EDB-FN) were also not altered (Supplementary Figure S4). This data suggests that TG2 modulation of actin cytoskeleton and Akt signaling also contributes to increased adipogenesis.

### *Tgm2*−/− adipose tissue display increased adipocyte number

**Figure 8:**
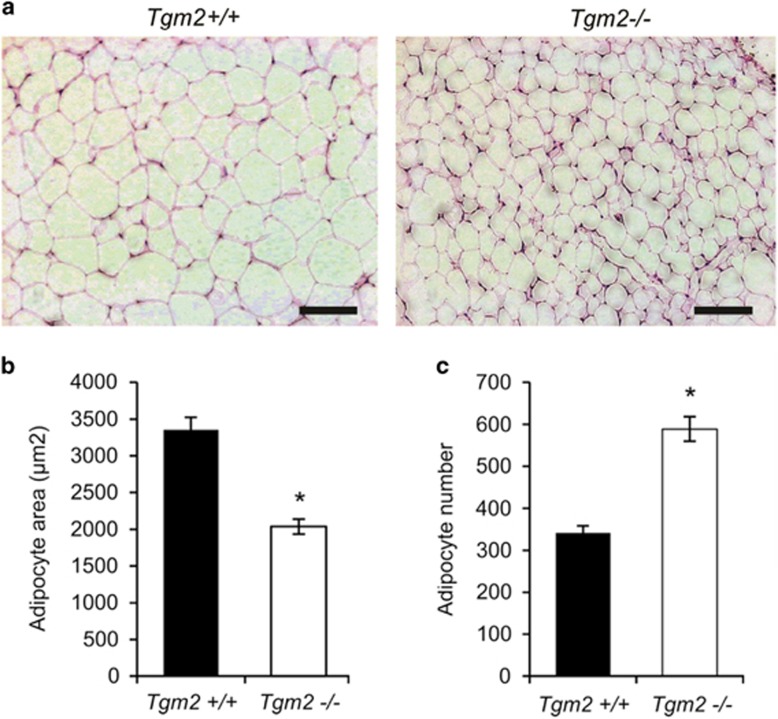
Increased adipocyte number in *Tgm2*−/− mouse WAT. (**a**) H&E stained sections of epididymal fat pads from *Tgm2*−/− and *Tgm2+/+* mice at 24 weeks of age. (**b**) Average adipocyte area, shows a significant decrease in the adipocyte area in *Tgm2*−/− compared with *Tgm2+/+* mice. (**c**) Average adipocyte number was significantly increased in *Tgm2*−/− mice compared with *Tgm2+/+.* Results are mean values±SEM (*n*=3), *P<0.05. Scale bar equals 100 *μ*m

To see how an increase in adipogenesis in *Tgm2*−/− MEFs *in vitro* translates to adipose tissue in mice, epididymal fat pad of *Tgm2*−/− and *Tgm2+/+* mice were used to assess adipocyte size and number *in vivo*. Figures 8a and b show that the adipocyte size is significantly reduced in *Tgm2*−/− mice compared with *Tgm2+/+* mice. However, adipocyte number is significantly increased in *Tgm2*−/− mice compared with *Tgm2+/+* mice (Figure 8c). The increase in adipocyte number in *Tgm2*−/− mice suggest increased proliferation of precursor cells and/or preadipocytes (hyperplasia), and their differentiation into mature adipocytes (adipogenesis).

## Discussion

Our previous work identified two members of TG family, FXIII-A and TG2, in WAT and demonstrated that FXIII-A can regulate adipocyte proliferation via promoting plasma FN matrix assembly which inhibits adipogenesis.^10^ In this study, we have examined the role of TG2 in adipogenesis and report for the first time that TG2 also acts as an inhibitor of adipocyte differentiation. We show that *Tgm2*−/− MEFs display accelerated and enhanced adipogenesis which is associated with downregulation of multiple anti-adipogenic signaling pathways that jointly lead to increased expression and activation of master transcription factors of adipogenesis, *Pparγ* and *Cebpα*, and increased lipid accumulation in the cells. The pathways identified in this study are; regulation of Pref-1 protein levels, β-catenin signaling and modulation of ROCK-mediated cytoskeletal tension and Akt signaling.

The identification of TG2 in as an anti-adipogenic factor is not entirely unexpected as TG2 has been implicated in regulation of number of pathways in multiple location in cells.^11^ Our report for the first time show that in MEFs *Tgm2* deficiency dramatically reduces *Pref-*1 protein expression. Pref-1 is an major inhibitor of adipocyte differentiation and whose expression is highest during early differentiation and then gradually disappears as the cells differentiate into adipocytes.^39,27^ Pref-1 protein mediates its effects on adipocyte differentiation by directly binding to FN, which activates integrin signaling to engage the MAPK/ERK pathway. This induces Sox9 expression which inhibits adipocyte differentiation.^29, 40^ *Pref-*1 knockout mice have increased adipose tissue mass, pre- and postnatal growth retardation and skeletal abnormalities^41^ and conversely *Pref-*1 overexpressing mice have reduced adipose tissue mass, impaired glucose tolerance and reduced insulin sensitivity.^42, 43^ Other effects of Pref-1 protein are also mediated by Sox9 which promotes chondrogenic commitment of MSCs, but inhibits chondrocyte maturation and osteoblast differentiation.^44^ Linked with Pref-1 function in chondrocyte maturation, TG2 has been shown to regulate the transition into the prehypertrophic stage during chondrocyte maturation. Premature, forced expression of TG2 accelerated progression toward prehypertrophy and it was shown that extracellular TG2 can increase Sox9 expression.^19^ Based on our work, it is highly possible that the effects of TG2 on chondrocytes may also be mediated via Pref-1. TG2 is also expressed by osteoblasts where it is located on the cell surface.^17^ TG2 knockout mice do not show any chondrogenic or osteogenic abnormalities during development or postnatally,^45^ which is likely due to compensatory function from upregulation of FXIII-A and TGFβ1.^46^ It is possible that TG2, jointly with other TG enzymes and Pref-1 may act as an upstream regulators of mesenchymal stem cell differentiation into the different lineages, particularly into chondrocytes, osteoblasts and adipocytes.

In addition to Pref-1 we reported here that TG2 regulates β-catenin nuclear translocation in preadipocytes. Canonical Wnt signaling is a crucial pathway that regulates lineage determination of MSCs. In preadipocytes, Wnt signaling maintains preadipocytes in undifferentiated state by inhibiting PPARγ and C/EBPα.^32^ During early phase of adipogenesis PPARγ suppresses Wnt signaling by increasing β-catenin degradation and PPARγ upregulation coincides with decreased total and nuclear β-catenin levels, suggesting a reciprocal relation between Wnt and PPARγ.^47, 48, 49^ Here we report that *Tgm2*−/− cells display increase in *Pparγ* and *Cebpα* mRNA expression and reduced β-catenin nuclear accumulation during the early phase of adipogenesis. When exogenous TG2 was added, a significant decrease in lipid accumulation was seen and this was associated with an increase in nuclear accumulation of β-catenin as well as decreased *Pparγ* and *Cebpα* mRNA expression. Interestingly, *Pref*-1 was shown to be a Wnt target gene and was, in fact, shown to be downregulated by TCF/β-catenin complex in fetal lung epithelial cells and MEFs.^50, 51, 52^ Furthermore, Pref-1 can act as a noncanonical Notch ligand and inhibit Notch signaling and, in turn, Wnt/β-catenin signaling is negatively regulated by Notch.^53^ This crosstalk between Notch and Wnt signaling may be one of the regulatory mechanism for Pref-1 production. We are currently exploring the mechanisms how TG2 affects the Pref-1 protein regulation.

It is well documented that canonical Wnt signaling inhibits adipogenesis and promotes osteogenesis in MSCs.^54^ Canonical Wnt signaling can also inhibit adipogenesis in lineage committed preadipocytes.^32^ Furthermore, canonical Wnt receptor LRP6 knockout MEFs show increased adipogenesis.^55^ In smooth muscle cells, extracellular TG2 regulates canonical Wnt signaling and β-catenin nuclear translocation which promotes calcification of the cell culture system. This effect in smooth muscle cells is mediated by extracellular TG2 binding to LRP5/6 on the smooth muscle cell surface and this reported interaction does not require transamidation activity.^30^ In this study, we show that extracellular TG2 activates canonical Wnt signaling contributing to the inhibitory effect on adipogenesis. While we did not address the exact mechanism how exogenous extracellular TG2 here regulates β-catenin nuclear translocation, it is plausible that the mechanism is the same as in smooth muscle cells. It is also possible that the exogenous TG2 promotes β-catenin release from the plasma membrane E-cadherin to the cytosol and from there to nucleus. This concept is supported by the observation that exogenous TG2 addition caused an increase in cytosolic and nuclear pools of β-catenin without affecting the total β-catenin levels in protein extracts.

Consistent with previous work on the role of TG2 in maintaining cytoskeletal tension, we also report here that *Tgm2*-deficient MEFs have decreased ROCK kinase activity and decreased actin stress fibers, which also contributes to increased adipogenesis. Adipogenesis is characterized by change in cell shape, from spindle-shaped preadipocytes to round adipocytes and this transition is partly determined by the cytoskeletal tension. During adipogenesis filamentous actin from stress fibers is rearranged to cortical pattern and down regulation of ROCK kinase disrupts actin stress fibers.^6, 56^ Inhibition of ROCK kinase was shown to promote adipogenesis and Akt signaling^35, 57^ and conversely activating ROCK kinase inhibits adipogenesis.^56^ TG2 was reported to activate ROCK via two pathways - retinoic acid-induced TG2 enzymatic activity was reported to activate ROCK kinase by intracellular TG2^58^ and cell surface TG2 was reported to amplify integrin mediated signaling to activate ROCK kinase in a non-enzymatic manner.^34^ ECM quantity and quality is a major regulator of cytoskeleton^59^ and it is known that cell surface TG2 cooperates with α5β1 integrins to enhance FN-integrin binding which is required for FN assembly. Furthermore, TG2 has been suggested to stabilize ECM in a number of studies and factors such as TGFβ—an inhibitor of adipogenesis—was shown to increase cell surface expression of TG2 and increase FN assembly.^60^ FN matrix itself is a major inhibitor of adipogenesis and must be decreased for the preadipocytes to allow differentiation toward mature adipocytes. However, in our study, we show that the absence of TG2 does not affect FN matrix levels or solubility in preadipocytes which strongly suggests that TG2 is not involved in FN matrix assembly and that cytoskeletal alterations in *Tgm2*−/− MEFs are not mediated by ECM itself, but likely via cell surface TG2 and the manner cells adhere to ECM. Indeed, *Tgm2*−/− deficient fibroblasts have been demonstrated to have an adhesion defect.^61, 62^ Moreover, the data strongly suggest that TG2 is not involved in MEF matrix assembly and does not appear to participate in MEF extracellular transamidation/crosslinking events. This is also supported by the fact that *Tgm2*−/− and *Tgm*2+/+ MEFs had similar levels of TG activity – this activity likely deriving from FXIII-A. Interestingly, both TG2 and FXIII-A act as negative regulators of adipogenesis and thus they may have a complementary effect on adipogenesis.

Increased fat mass in obesity is associated with an increase in adipocyte cell size and/or adipocyte number which are reactions to expand adipose tissue upon need to increase energy storage.^3^ Defects in this expansion are linked to obesity-linked comorbidities such as development of type 2 diabetes.^4, 5^ In this work, we have identified a new factor, TG2 that maintains preadipocyte state and thus acts as a negative regulator of adipogenesis. It is thus likely that regulation of TG2 in preadipocytes is tightly controlled to balance proliferation and differentiation. Further understanding of TG2 and its role and regulation in metabolic disorders would aid the development of new therapies to maintain healthy energy metabolism.

## Materials and Methods

### Animals

*Tgm2*−/− mice were described before.^45^ Wild-type (WT) mice were purchased from Jackson Laboratories (Bar Harbor, Maine, USA). Mice were kept under a normal diurnal cycle in a temperature-controlled room and fed with standard chow. Animal procedures (WAT extraction and MEF isolation) and study protocols were approved by the McGill University Animal Care Committee.

### Antibodies and proteins

Antibodies against rabbit anti-Akt (pan), rabbit anti- phospho-Akt (Ser^473^)(D9E), rabbit anti-PPARγ, rabbit anti-histone, rabbit anti-Pref-1 were purchased from Cell Signaling Technology Inc (Beverly, MA, USA). Rabbit anti-actin, mouse anti-tubulin antibodies were obtained from Sigma-Aldrich (St Louis, MO, USA). Rabbit anti-fibronectin antibody, human recombinant MYPT1 (654–880) and rabbit anti-phospho-MYPT1 (Thr696) were from EMD Millipore (Billerica, MA, USA). Rabbit anti-C/EBPα purchased from Santa Cruz Biotechnology (Santa Cruz, CA, USA). Rabbit anti-*β*-catenin purchased from Abcam (Cambridge, MA, USA). Mouse monoclonal TG2 Ab-3 antibody (Clones CUB 7402+TG100) was from Fisher Scientific (Fremont, CA, USA). Horseradish peroxidase-conjugated anti-rabbit IgG was purchased from Cell Signaling Technology Inc. Horseradish peroxidase-conjugated goat anti-rabbit IgM was purchased from Santa Cruz Biotechnology. Horseradish peroxidase-conjugated anti-mouse, and anti-rabbit IgG were from Jackson ImmunoResearch Inc. Alexa Fluor 488 and 596, Alexa Fluor 568-phalloidin and Bodipy 493/503 were from Life Technologies (Grand Island, NY, USA).

### Reagents

Dulbecco's modified Eagle's medium (DMEM) and 0.5 mg/ml trypsin and 0.2 mg/ml EDTA from ATCC (Cedarlane, ON, Canada). Fetal bovine serum (FBS) and penicillin-streptomycin were from Gibco (Burlington, ON, Canada). Oil Red O, IGEPAL CA-630, dexamethasone, insulin, 3-Isobutyl-1-methylxanthine (IBMX), 3,3′,5,5′-Tetramethylbenzidine (TMB) were from Sigma-Aldrich. Troglitazone were purchased from Santa Cruz Biotechnology. Sulfo-NHS-LC-biotin and 5-(biotinamido)pentylamine were purchased from Pierce (Rockford, IL, USA). ECL kit was from Zmtech Scientifique (Montreal, QC, Canada). All other reagents unless otherwise specified were purchased from Sigma-Aldrich or Fisher Scientific.

### MEF cell culture and differentiation

Mouse embryonic fibroblasts (MEFs) were prepared from 13.5 days *Tgm2+/+* and *Tgm2*−/− mouse embryos, MEFs isolation, culture and staining with Oil Red O was done according to previously published protocol.^10^ MEFs were differentiated into adipocytes with 10% FBS, 1*μ*M dexamethasone, 0.5 mM isobutyl-1-methylzanthine, 1 *μ*g/ml insulin, and 10*μ*M troglitazone for 2 days. On day 2, media were replaced with maintenance medium which includes 10% FBS and 1 *μ*g/ml insulin and 10*μ*M troglitazone. On day 4, maintenance media was replaced with medium containing 10% FBS and cells were cultured in this until the end of the experiment, that is, day 8. Intracellular triglyceride was stained with Oil Red O and quantified; cells were counter stained with haematoxylin and photographed with a light microscope.

### Whole-mount staining, immunofluorescence and histology

For whole-mount staining, mouse WAT from *Tgm2*+/+ mice was fixed in 10% neutral-buffered formalin. Fixed tissue was cut with a scalpel to 5 mm × 5 mm sections, and blocked with 3% BSA, 0.3% Triton X-100, in PBS for 12-24 h at 4 ^°^C. Tissue pieces were incubated with primary antibodies overnight at 4 °C which was followed by incubation with Alexa Fluor-conjugated secondary antibodies for 1 h at room temperature. Nuclei were stained with DAPI. Antibody omission and isotype specific immunoglobulins were used as controls.^10^ For histology, mouse epididymal fat pad from *Tgm2*+/+ and *Tgm2*−/− mice were fixed in 10% neutral-buffered formalin and paraffin embedded and stained with eosin and hematoxylin.^10^ For immunofluorescence, cells were grown in 8-well Nunc Lab-Tek II glass chamber slides (Fisher Scientific). Cells were fixed with 10% neutral-buffered formalin for 30 min at room temperature or with 1% formaldehyde for 15 min at room temperature (for optimal cell-surface protein staining). Staining was done as previously described.^10^ Quantification was done with Image J (v1.34i, NIH, Bethesda, MD, USA).

### Protein extraction and western blotting

Total cell lysate was prepared with lysis buffer containing 20 mM Tris-HCl, pH 7.5, 5 mM EDTA, 150 mM NaCl, 0.5% DOC, 0.5% Triton X-100, 1 mM PMSF and 1 mM orthovanadate and protease inhibitor cocktail (Sigma). Cell lysates were incubated on ice for 30 min with occasional vortexing and then centrifuged for 15 min at 15,000x*g* at 4 °C. Nuclear and cytosolic fractions were prepared as previously described.^63^ Deoxycholate (DOC)-soluble and DOC-insoluble FN matrix extracts were prepared as described previously.^64^ Western blotting and quantification of bands with Image J (v1.34i, NIH) was done as previously described.^10^

### Cell surface biotinylation

Cell surface biotinylation was done for *Tgm2+/+* MEFs as previously described.^10^

### RT-PCR and Real-time PCR

mRNA was isolated using Trizol method. RNA was treated with DNase (New England Biolabs, Ipswich, MA, USA), and PCR was performed with SuperScriptIII One-Step RT-PCR System with Platinum Taq DNA Polymerase (Invitrogen, Burlington, ON, Canada). PCR products were analyzed by 1.5% agarose gel electrophoresis. Primers used were previously described *Pref-1,*^65^ EDA, EDB,^66^ *Fn*, *Gapdh*,^17^ *Pparγ2* and *Cebpα*.^67^ Real-time PCR was performed on a ABIHT7900 RT-PCR machine using the comparative C_T_ method in triplicate using the TaqMan Universal Master Mix II. Expression levels of *Pparγ2* (Mm 01184322_m1, *Cebpα* (Mm 514283_s1) and normalized to *Rn18S* (Mm 03928990_g1).

### *In situ* transglutaminase activity assay

*In situ* transglutaminase activity assay was done by giving 2 mM 5-(biotinamido)pentylamine to the cells during differentiation. At the indicated time point, total cell extracts were prepared with 50 mM Tris-HCl (pH 8.0), 135 mM NaCl, and 1% Triton X-100, 1 mM EDTA, 1 mM sodium orthovanadate and EDTA-free protease inhibitor cocktail. To see the basal level of TG activity under these conditions, biotinamidopentylamine (BPA) was added to the cultures on day -1 (i.e., 1 day after the cells are confluent). The total cell lysate was extracted on day 0, that is, 24 hr after the BPA was added, but before adipogenic treatment was started. The value obtained by microplate TG-activity assay from cell lysate without BPA was subtracted from the value of cell lysate containing BPA, and the resulting value was considered TG-activity on day 0. Microplate assay to detect biotin was done as previously described.^10^

### ROCK kinase activity

ROCK kinase activity was determined by using microplate *in vitro* kinase assay as previously described.^10^ Cell lysate was prepared with 50 mM Tris-HCl, pH 7.5, 150 mM NaCl, 1 mM 2-glycerophosphate, 1% Triton X-100, 1 mM EDTA, 1 mM EGTA, 1 mM Na_3_VO_4_ and EDTA-free protease inhibitors cocktail.

### Statistical analysis

All values are expressed as standard error of the mean (SEM) of three independent experiments. Statistical significance was assessed by student's T-test. *P*-values are as follows: *P>0.05, **P>0.01, ***P>0.001.

## Abbreviations

TG2: Transglutaminase 2
WAT: white adipose tissue
ECM: extracellular matrix
FN: fibronectin
COL I: type I collagen
FXIII-A: Factor XIII-A
*Tgm2*+/+: TG2 wild type
*Tgm2*−/−: TG2 deficient mice
MEFs: mouse embryonic fibroblasts
exoTG2: exogenous TG2 enzyme
Pref-1: preadipocyte factor 1
RhoA: Ras homolog gene family, member A
ROCK: Rho-associated, coiled-coil containing protein kinase
PPARγ: Peroxisome proliferator-activated receptor gamma
C/EBPα: CCAAT/enhancer-binding protein alpha
MAPK: mitogen-activated protein kinases
ERK: extracellular signal-regulated kinases
MSCs: mesenchymal stem cells
TGFβ1: transforming growth factor beta
LRP: Low-density lipoprotein receptor-related protein
DOC: Deoxycholate
DMEM: Dulbecco's modified Eagle's medium
FBS: Fetal bovine serum
IBMX: 3-Isobutyl-1-methylxanthine
TMB: 3,3′,5,5′-Tetramethylbenzidine
SEM: standard error of the mean
