# Supplementary figures and images for: Transglutaminase 2—a novel inhibitor of adipogenesis
Source: Cell Death Dis. 2015 Aug 27;6(8):e1868–. doi: 10.1038/cddis.2015.238 (PMC4558519; doi:10.1038/cddis.2015.238)

# Supplemental Figures

## Fig. S1

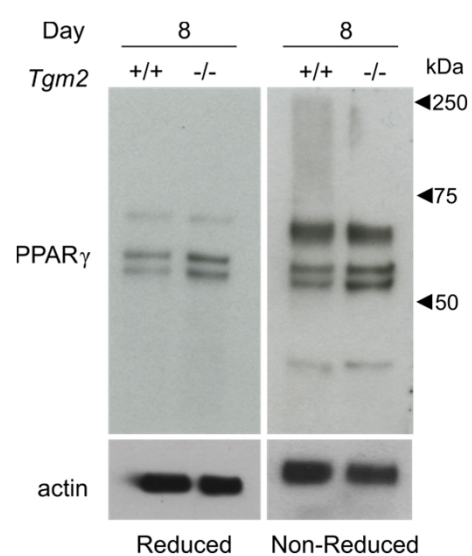

Supplement: Supplementary Figure 1 [file cddis2015238x1.pdf]

## Supplemental Figures

### Fig. S2

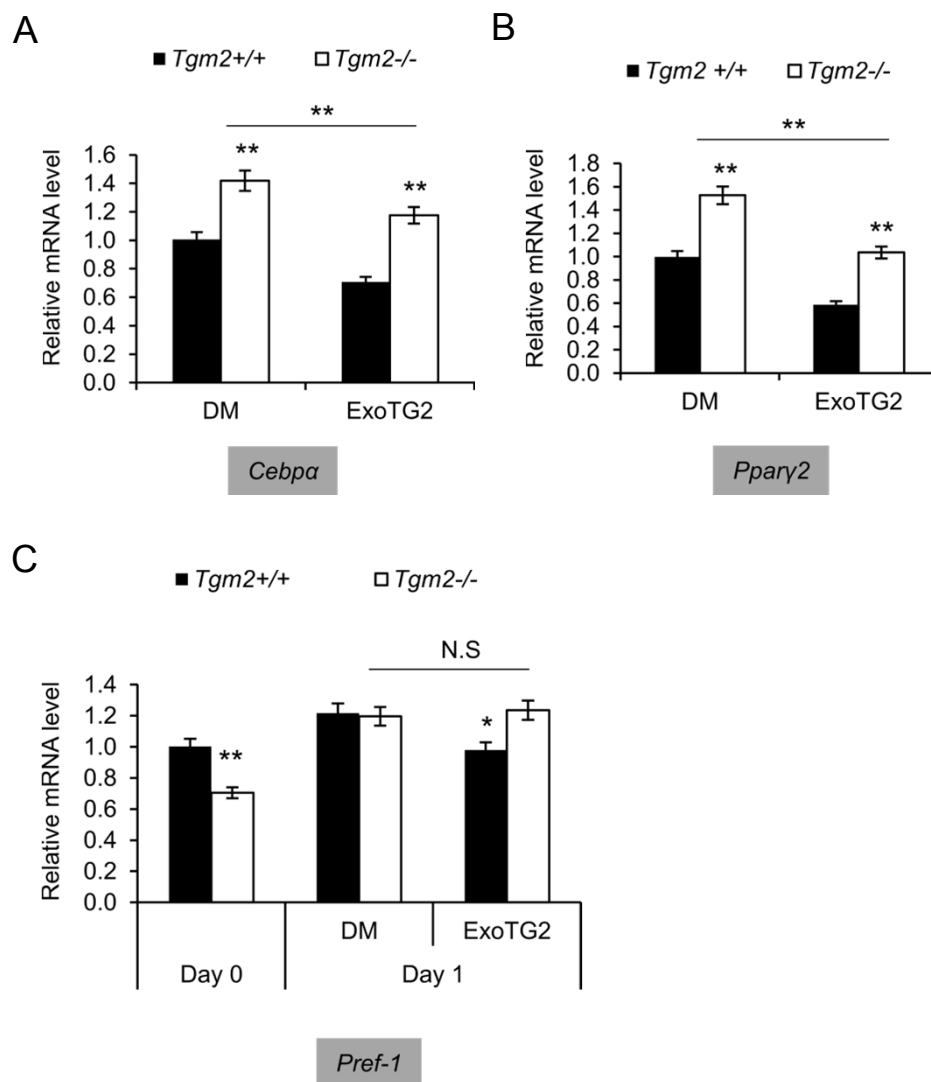

Supplement: Supplementary Figure 2 [file cddis2015238x2.pdf]

# Supplemental Figures

## Fig. S3

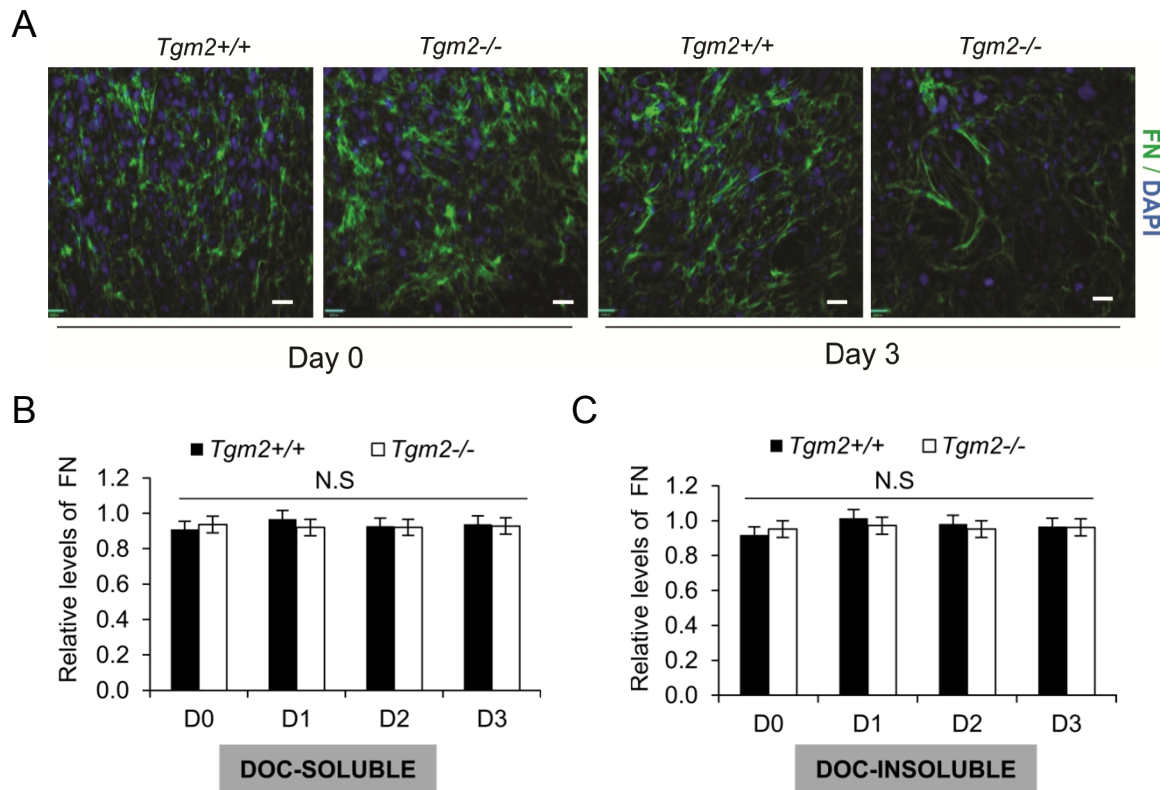

Supplement: Supplementary Figure 3 [file cddis2015238x3.pdf]

# Supplemental Figures

## Fig. S4

A

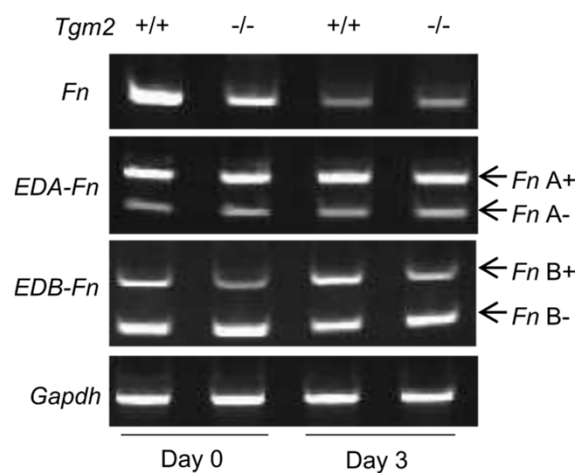

B

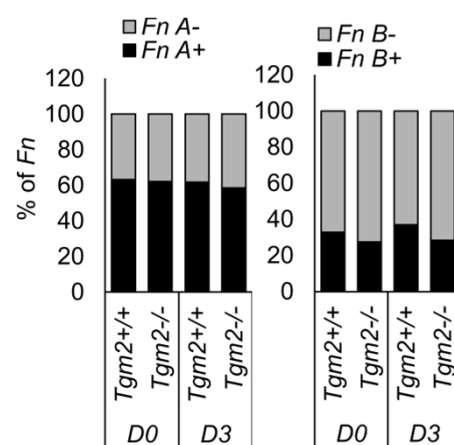

Supplement: Supplementary Figure 4 [file cddis2015238x4.pdf]
