# Supplementary material for: Transglutaminase 2—a novel inhibitor of adipogenesis
Source: Cell Death Dis. 2015 Aug 27;6(8):e1868–. doi: 10.1038/cddis.2015.238 (PMC4558519; doi:10.1038/cddis.2015.238)
Supplement: Supplementary Figure Legends [file cddis2015238x5.doc]

**Fig. S1. PPARγ is not found in high molecular weight forms in MEFs and thus not crosslinked by TG2 during adipocyte differentiation.** Western blot analysis of total cell lysate on day 8 in both reduced and non-reduced conditions for PPARγ protein, higher molecular weight products expected to be found at molecular weights between 75 to 250 kDa. No higher molecular weight products were observed in either condition.

**Fig. S2. Extracellular TG2 down regulates mRNA expression of *Pparγ* and *Cebpα*. (A,B)** Quantification of mRNA analyses from **Fig 6C** for *Pparγ* and *Cebpα* and normalized to *Gapdh***,** show significant reduction in *Pparγ* and *Cebpα* in both *Tgm2*-/- and *Tgm2*+/+ MEFs. Error bars ± SEM; N.S-Not Significant; *p<0.05; **p<0.01. **(C)** Quantification of mRNA analyses from **Fig 6G** for *Pref-1* normalized to *Gapdh*, shows no difference in *Tgm2*-/- MEFs, but surprisingly show a significant reduction in *Tgm2+/+* MEFs.

**Fig. S3. FN levels were not altered in *Tgm2-/-* MEFs. (A)** Immunofluorescence staining for FN in *Tgm2+/+* and *Tgm2-/-* MEFs on day 0 and day 3. No major change was observed in FN matrix levels. Scale bar equals 200µm. **(B)** Quantification of FN in deoxycholate (DOC)-soluble and DOC-insoluble fractions after Western blotting, shows no changes in FN solubility. Quantification of FN Western blots was done by normalizing to loading controls. Actin was used for DOC-soluble and vimentin for DOC-insoluble fractions. N.S-Not Significant; Error bars ± SEM (n=3).

**Fig. S4. *Fn* and cellular *Fn* expression did not change in *Tgm2*-/- MEFs. (A)** mRNA expression of *Fn* and *EDA-Fn* and *EDB-Fn* in *Tgm2+/+* and *Tgm2-/-* MEFs on day 0 and day 3. On day 0, a decrease in *Fn* expression is seen in *Tgm2*-/- cells, however, the difference disappears on day 3. *EDA-Fn* and *EDB-Fn* shows no changes. **(B)** Quantification of RT-PCR of *EDA-Fn* and *EDB-Fn* expressed as percentage of *Fn* on day 0 and day 3. No changes are seen.
